# Supplementary material for: Singlet fission for quantum information and quantum computing: the parallel JDE model
Source: Sci Rep. 2020 Oct 28;10:18480. doi: 10.1038/s41598-020-75459-x (PMC7595132; doi:10.1038/s41598-020-75459-x)
Supplement: Supplementary file 1 — Supplementary Information [file 41598_2020_75459_MOESM1_ESM.pdf]

Supporting information for: Singlet fission for quantum information and quantum computing: The parallel *JDE* model

Kori Smyser and Joel Eaves*

Department of Chemistry, University of Colorado. Boulder, Colorado 80309-0215 United States

Contents

S1 Deriving the *JDE* model **S1**

S2 Matrix elements of the *JDE* model in zero field **S2**

S3 Zero-field selection rules **S2**

S4 Matrix elements of the *JDE* model in a Zeeman field **S3**

S5 Zeeman-field selection rules **S3**

S6 The $B||x'$ hamiltonian **S5**

S7 Simulating spectra **S6**

S1 Deriving the *JDE* model

We start with the general, bilinear hamiltonian that describes all of the interactions between electrons $1-4$ in orbitals i and j (equation 5, Fig. 2a),

$$\mathcal{H} = \sum_{\substack{i,j=1 \\ i\neq j, i>j}}^{4} \mathbf{s}_i \cdot \mathbf{O}_{ij} \cdot \mathbf{s}_j, \tag{S1}$$

where $\mathbf{s}$ is the electron spin operator and $\mathbf{O}$ is a dyadic tensor containing all spin-spin interactions. We then expand this sum and collect terms,

$$\begin{aligned}\mathcal{H} &= \mathbf{s}_1 \cdot \mathbf{O}_{12} \cdot \mathbf{s}_2 + \mathbf{s}_1 \cdot \mathbf{O}_{13} \cdot \mathbf{s}_3 + \mathbf{s}_1 \cdot \mathbf{O}_{14} \cdot \mathbf{s}_4 + \mathbf{s}_2 \cdot \mathbf{O}_{23} \cdot \mathbf{s}_3 + \mathbf{s}_2 \cdot \mathbf{O}_{24} \cdot \mathbf{s}_4 + \mathbf{s}_3 \cdot \mathbf{O}_{34} \cdot \mathbf{s}_4 \\ &= \mathbf{s}_1 \cdot \mathbf{O}_{13} \cdot \mathbf{s}_3 + \mathbf{s}_1 \cdot \mathbf{O}_{14} \cdot \mathbf{s}_4 + \mathbf{s}_2 \cdot \mathbf{O}_{23} \cdot \mathbf{s}_3 + \mathbf{s}_2 \cdot \mathbf{O}_{24} \cdot \mathbf{s}_4 + \mathbf{s}_1 \cdot \mathbf{O}_{12} \cdot \mathbf{s}_2 + \mathbf{s}_2 \cdot \mathbf{O}_{24} \cdot \mathbf{s}_4 \\ &= \mathbf{s}_1 \cdot \mathbf{O}_{13} \cdot \mathbf{s}_3 + \mathbf{s}_1 \cdot \mathbf{O}_{14} \cdot \mathbf{s}_4 + \mathbf{s}_2 \cdot \mathbf{O}_{23} \cdot \mathbf{s}_3 + \mathbf{s}_2 \cdot \mathbf{O}_{24} \cdot \mathbf{s}_4 + H_A + H_B.\end{aligned} \tag{S2}$$

Here, $H_A = \mathbf{s}_1 \cdot \mathbf{O}_{12} \cdot \mathbf{s}_2$ and $H_B = \mathbf{s}_3 \cdot \mathbf{O}_{34} \cdot \mathbf{s}_4$.

*Email: joel.eaves@colorado.edu

Next, we make the assumption that all inter-chromophore couplings are equivalent, so that $\mathbf{O}_{13} = \mathbf{O}_{14} = \mathbf{O}_{23} = \mathbf{O}_{24} \equiv \mathbf{O}$. This allows us to group terms and substitute the chromophore spin, $\mathbf{S}_A = \mathbf{s}_1 + \mathbf{s}_2$ for chromophore A and $\mathbf{S}_B = \mathbf{s}_3 + \mathbf{s}_4$ for chromophore B. With this approximaton the hamiltonian becomes

$$\begin{aligned} \mathcal{H} &= \mathbf{s}_1 \cdot \mathbf{O} \cdot \mathbf{s}_3 + \mathbf{s}_1 \cdot \mathbf{O} \cdot \mathbf{s}_4 + \mathbf{s}_2 \cdot \mathbf{O} \cdot \mathbf{s}_3 + \mathbf{s}_2 \cdot \mathbf{O} \cdot \mathbf{s}_4 + H_A + H_B \\ &= \mathbf{s}_1 \cdot \mathbf{O} \cdot (\mathbf{s}_3 + \mathbf{s}_4) + \mathbf{s}_2 \cdot \mathbf{O} \cdot (\mathbf{s}_3 + \mathbf{s}_4) + H_A + H_B \\ &= (\mathbf{s}_1 + \mathbf{s}_2) \cdot \mathbf{O} \cdot (\mathbf{s}_3 + \mathbf{s}_4) + H_A + H_B \\ &= \mathbf{S}_A \cdot \mathbf{O}_{AB} \cdot \mathbf{S}_B + H_A + H_B \\ &= H_{AB} + H_A + H_B, \end{aligned} \tag{S3}$$

where $H_{AB} = \mathbf{S}_A \cdot \mathbf{O}_{AB} \cdot \mathbf{S}_B$.

The rank-2 Cartesian tensors $\mathbf{O}_{ij}$ have matrix representations which are reducible. They may be written as a sum of three terms corresponding to their irreducible parts,[1]

$$\mathbf{O}_{ij} = \frac{\mathrm{Tr}\left(\mathbf{O}_{ij}\right)}{3}\mathbb{1} + \frac{1}{2}\left(\mathbf{O}_{ij} - \mathbf{O}_{ji}\right) + \left(\frac{1}{2}\left(\mathbf{O}_{ij} + \mathbf{O}_{ji}\right) - \frac{\mathrm{Tr}\left(\mathbf{O}_{ij}\right)}{3}\right). \tag{S4}$$

The first term is a scalar (rank-zero) and is rotationally invariant (isotropic); the second term is an anti-symmetric rank-one tensor and the third is a rank-two anisotropic, symmetric and traceless tensor. Each term can be conveniently represented as a spherical tensor - a property that we exploit when finding matrix elements with angular momentum states.

Following the simplifications in the main text, we discard the inter-chromophore anisotropic exchange interaction and reduce the hamiltonian by setting $\mathbf{O}_{AB} = \frac{\mathrm{Tr}(\mathbf{O}_{AB})}{3} \equiv J$. The intra-chromophore interaction tensors become $\mathbf{O}_{12} = \mathbf{O}_{34} \equiv \mathbf{D}$. $\mathbf{D}$ is the zero-field spin-dipole tensor for a single chromophore. When the chromophores share principal axes, the hamiltonian in the principal frame of the $\mathbf{D}$ tensor becomes the parallel *JDE* model from the main text,

$$\mathcal{H} = J\,\mathbf{S}_A \cdot \mathbf{S}_B + H_{ZFS}. \tag{S5}$$

which, in the principal frame of $\mathbf{D}$, simplifies to equation 6.[2]

S2 Matrix elements of the *JDE* model in zero field

The isotropic term is rotationally invariant. As a result, it is diagonal in the basis of total spin angular momentum, $|S, M\rangle$. The nontrivial terms are from H_{ZFS}. To evaluate H_{ZFS}, we write it in its spherical tensor form and take matrix elements. The result is

$$\begin{aligned} \langle S', M'|\mathcal{H}|S, M\rangle &= \frac{J}{2}\left(S\left(S+1\right) - 4\right)\delta_{S,S'}\delta_{M,M'} \\ &\quad + \sum_{q=-2}^{+2}(-1)^q D^{(2)}_{-q}\left(\langle S', M'|S^{(2)}_{A,q}|S, M\rangle + \langle S', M'|S^{(2)}_{B,q}|S, M\rangle\right). \end{aligned} \tag{S6}$$

Where $S^{(2)}_{A,q}$, for example, is the spherical tensor representation of the q-th spherical component of the dyad formed by taking the tensor product of $\mathbf{S}_A$ with itself. The sum goes over spherical tensor components q and in terms of the spectroscopic parameters, D and E, the three non-zero spherical tensor components of $\mathbf{D}$ are $D^{(2)}_0 = \sqrt{\frac{2}{3}}D$, $D^{(2)}_{-2} = E$ and $D^{(2)}_{+2} = E$. As a result of the Wigner-Eckhart theorem, the remaining matrix elements inside the sum can be expressed in terms of 3-j symbols and reduced matrix elements.[3] The selection rules on S, S', M, M' come from the 3-j symbols. At zero field the quantization axis lies along the principal axis of the $\mathbf{D}$ tensor.

S3 Zero-field selection rules

Because the hamiltonian for parallel, identical chromophores, is invariant to exchanging A with B, transitions between spin states with different eigenvalues of the exchange operator are forbidden. The exchange symmetry forbids any transitions into the $|{}^3TT\rangle$ states from either $|{}^1TT\rangle$ or the $|{}^5TT\rangle$ states.

To apply perturbation theory we rewrite the hamiltonian as the sum of a spin-conserving reference hamiltonian, $H_0 = J\mathbf{S}_A \cdot \mathbf{S}_B + D\left(S_z^2 - \vec{S}^2/3\right)$, and a perturbation, $V = -\frac{2D}{3}\left(2S_{Az}S_{Bz} - S_{Ax}S_{Bx} - S_{Ay}S_{By}\right) - 2E\left(S_{Ax}S_{Bx} - S_{Ay}S_{By}\right) + \frac{E}{2}\left(S_+^2 + S_-^2\right)$. Motivated by degenerate perturbation theory and the form of V in this representation, we choose the symmetric and anti-symmetric linear combinations of the $|S, M\rangle$ states as our zero-field basis. For the quintet states, these are,

$$
\begin{aligned}
|Q_{x^2-y^2}\rangle &= \frac{1}{\sqrt{2}}\left(|^5TT_{+2}\rangle + |^5TT_{-2}\rangle\right) = \frac{1}{\sqrt{2}}\left(|xx\rangle - |yy\rangle\right) \\
|Q_{xy}\rangle &= \frac{1}{\sqrt{2}}\left(|^5TT_{+2}\rangle - |^5TT_{-2}\rangle\right) = \frac{i}{\sqrt{2}}\left(|xy\rangle + |yx\rangle\right) \\
|Q_{xz}\rangle &= \frac{1}{\sqrt{2}}\left(|^5TT_{+1}\rangle - |^5TT_{-1}\rangle\right) = -\frac{1}{\sqrt{2}}\left(|xz\rangle + |zx\rangle\right) \\
|Q_{yz}\rangle &= \frac{1}{\sqrt{2}}\left(|^5TT_{+1}\rangle + |^5TT_{-1}\rangle\right) = -\frac{i}{\sqrt{2}}\left(|yz\rangle + |zy\rangle\right) \\
|Q_{z^2}\rangle &= |^5TT_0\rangle = \frac{1}{\sqrt{6}}\left(2|zz\rangle - |xx\rangle - |yy\rangle\right).
\end{aligned}
\tag{S7}
$$

As required by degenerate perturbation theory, all splittings between $\pm M$-levels in the reference hamiltonian smoothly go to zero as $V \to 0$.

The first term in V is of similar form to the $|Q_{z^2}\rangle$ state (equation S7) and couples it to the $|^1TT\rangle$ state by $|\langle ^1TT|V|Q_{z^2}\rangle| \sim D$; the second term couples the $|^1TT\rangle$ state to the $|Q_{x^2-y^2}\rangle$ state by $|\langle ^1TT|V|Q_{x^2-y^2}\rangle \sim E$; and the third term in V weakly mixes the non-degenerate quintet states, $|Q_{z^2}\rangle$ and $|Q_{x^2-y^2}\rangle$ so weakly that we ignore it. All other coupling elements go to zero.

S4 Matrix elements of the *JDE* model in a Zeeman field

For an applied Zeeman field so that the induced Zeeman splitings are large relative to H_{ZFS} but not with respect to J, it is natural to choose the quantization axis to lie along the polarization of the Zeeman field. This requires a "passive" rotation of the principal frame, in which the **D** tensor is diagonal, into the lab frame defined by the Zeeman field.[3]

The matrix elements of the *JDE* model in an applied field are

$$
\begin{aligned}
\langle S', M'|\mathcal{H}|S, M\rangle &= g\mu_B B_0 M \delta_{S',S}\delta_{M',M} + \frac{J}{2}\left(S\left(S+1\right) - 4\right)\delta_{S,S'}\delta_{M,M'} \\
&+ \sum_q \sum_{q'} (-1)^q \mathcal{D}^{(2)}_{q',-q}(\phi, \theta, \psi) D^{(2)}_{q'}\left(\langle S', M'|S^{(2)}_{A,q}|S, M\rangle + \langle S', M'|S^{(2)}_{B,q}|S, M\rangle\right),
\end{aligned}
\tag{S8}
$$

where $\mathcal{D}^{(2)}_{q',q}(\phi, \theta, \psi)$ is the (q', q)-th element of the Wigner D-matrix for the rotation by the three Euler angles, ϕ, θ and ψ, and $D^{(2)}_{q'}$ is the q'-th element of the ZFS tensor introduced in equation S5. As in Supplementary Note S2, we apply the Wigner-Eckart theorem to evaluate the matrix elements $\langle S', M'|S^{(2)}_{A,q}|S, M\rangle$ and $\langle S', M'|S^{(2)}_{B,q}|S, M\rangle$.[3]

S5 Zeeman-field selection rules

For field strengths relevant to the experiments we model, the behavior of the mixed adiabatic $|\alpha\rangle$ basis states, discussed in the main text, follows the diabatic $|S, M\rangle$ states very closely. The selection rules for transitions between $|S, M\rangle$ states are also realized in the application of the Wigner-Eckart theorem. It states that a rank-k, component-q tensor will connect state $|S, M\rangle$ to state $|S', M'\rangle$ if both the triangle inequality and the integer rule are satisfied. In our case, $\langle S', M'|V^{(k)}_q|S, M\rangle$ will be nonzero only when

1. $|S - k| \leq S' \leq S + k$, and

2. $\Delta M = q$.

The parallel *JDE* model is a sum of Zeeman, Exchange and ZFS interactions (equation S8). In the $|S, M\rangle$ basis, the Zeeman and Exchange interactions are rank-zero and therefore only connect a state with itself. The ZFS interaction is rank-two and all five of its components may be non-zero if the system is rotated with respect to the static field, B_0. Combining these rotational symmetry-based selection rules with the exchange selection rule discussed in Supplementary Note S3, we find that for parallel chromophores:

1. S=0 → S'=0,2
   S=1 → S'=1
   S=2 → S'=0,2, and

2. $\Delta M = 0,\ \pm 1,\ \pm 2$.

The analytical form for the coupling matrix elements between ^1TT and the ^5TT manifold, in the $|S, M\rangle$ basis are:

$$\begin{aligned}
\langle {}^1TT|V|{}^5TT_{-2}\rangle &= \frac{1}{\sqrt{3}}\mathrm{e}^{-2i\psi}\left(D\sin^2\theta + 2E\left(\mathrm{e}^{-2i\phi}\cos^4\frac{\theta}{2} + \mathrm{e}^{2i\phi}\sin^4\frac{\theta}{2}\right)\right)\\
\langle {}^1TT|V|{}^5TT_{-1}\rangle &= \frac{2}{\sqrt{3}}\mathrm{e}^{-i\psi}\sin\theta\left(D\cos\theta - E\left(\cos\theta\cos 2\phi - i\sin 2\phi\right)\right)\\
\langle {}^1TT|V|{}^5TT_{0}\rangle &= \frac{\sqrt{2}}{3}\left(D\left(3\cos^2(\theta) - 1\right) + 3E\sin^2(\theta)\cos(2\phi)\right)\\
\langle {}^1TT|V|{}^5TT_{+1}\rangle &= \frac{2}{\sqrt{3}}\mathrm{e}^{i\psi}\sin\theta\left(-D\cos\theta + E\left(\cos\theta\cos 2\phi + i\sin 2\phi\right)\right)\\
\langle {}^1TT|V|{}^5TT_{+2}\rangle &= \frac{1}{\sqrt{3}}\mathrm{e}^{2i\psi}\left(D\sin^2\theta + 2E\left(\mathrm{e}^{-2i\phi}\sin^4\frac{\theta}{2} + \mathrm{e}^{2i\phi}\cos^4\frac{\theta}{2}\right)\right)
\end{aligned} \tag{S9}$$

When $\psi = 0$, θ and ϕ correspond to the spherical polar and azimuthal angle between the lab and principal axes. It is simple to see that when $\theta = 0, \pm 90^\circ$ transitions from ^1TT into $S = 2, M = \pm 1$ are forbidden. Fig. 4 in the main text shows how the angular dependence of the coupling matrix elements manifests in the prompt EPR spectra.

S6 The $B||x'$ hamiltonian

Below is the analytical form of the hamiltonian for $B||x'$ in the $|S, M\rangle$ basis. Basis states are ordered with increasing S and M, across columns and down rows.

$$H_{B||x'} = \begin{pmatrix}
-2J & 0 & 0 & 0 & \frac{D+E}{\sqrt{3}} & 0 & -\frac{\sqrt{2}}{3}(D-3E) & 0 & \frac{D+E}{\sqrt{3}} \\
0 & -B_0 - J + \frac{D}{6} - \frac{E}{2} & 0 & \frac{-(D+E)}{2} & 0 & 0 & 0 & 0 & 0 \\
0 & 0 & -J - \frac{D}{3} + E & 0 & 0 & 0 & 0 & 0 & 0 \\
0 & \frac{-(D+E)}{2} & 0 & B_0 - J + \frac{D}{6} - \frac{E}{2} & 0 & 0 & 0 & 0 & 0 \\
\frac{D+E}{\sqrt{3}} & 0 & 0 & 0 & -2B_0 + J - \frac{D}{3} + E & 0 & \frac{D+E}{\sqrt{6}} & 0 & 0 \\
0 & 0 & 0 & 0 & 0 & -B_0 + J + \frac{D}{6} - \frac{E}{2} & 0 & \frac{D+E}{2} & 0 \\
-\frac{\sqrt{2}}{3}(D-3E) & 0 & 0 & 0 & \frac{D+E}{\sqrt{6}} & 0 & J + \frac{D}{3} - E & 0 & \frac{D+E}{\sqrt{6}} \\
0 & 0 & 0 & 0 & 0 & \frac{D+E}{2} & 0 & B_0 + J + \frac{D}{6} - \frac{E}{2} & 0 \\
\frac{D+E}{\sqrt{3}} & 0 & 0 & 0 & 0 & 0 & \frac{D+E}{\sqrt{6}} & 0 & 2B_0 + J - \frac{D}{3} + E
\end{pmatrix}$$

S7 Simulating spectra

To simulate spectra, we calculate the hamiltonian and find field values of B_0 which satisfy $\Delta E(B_0) = q\mu_B B_1$, where B_1 is the magnitude of the applied microwave frequency and $\Delta E(B_0)$ is the energy gap between $|\alpha\rangle$ states. We then generate a stick spectrum over the range of B_0. The intensities of transitions are then calculated, as discussed in the main text as, $I(B_0) = \sum_{\alpha,\beta} |\langle\alpha|S_x|\beta\rangle|^2(P_\alpha - P_\beta)\delta(\epsilon_\alpha - \epsilon_\beta)$. Finally, we convolve the stick spectrum with a Gaussian lineshape function of intensity $I(B_0)$.

References

1. Sakurai, J. J. & Tuan, S. F. *Modern quantum mechanics* (Addison-Wesley Pub. Co., Reading, Mass., 1994), Rev. edn.

2. Weil, J. A. & Bolton, J. R. *Electron Paramagnetic Resonance: Elementary Theory and Practical Applications* (John Wiley & Sons, Hoboken, N.J., 2007), 2nd edn.

3. Edmonds, A. R. *Angular momentum in quantum mechanics* (Princeton university press, 1996). We refer the reader to the 1996, revised edition of Edmonds's book and note that earlier editions contain errors. See Ref. 4 for more details.

4. Bouten, M. On the rotation operators in quantum mechanics. *Physica* **42**, 572–580 (1969).
